# In Silico Design of gRNA for CRISPR System for Detection of Multidrug Resistant Tuberculosis Using Indian Mycobacterium tuberculosis Genomes: A Computational Study

**DOI:** 10.7759/cureus.101851

**Published:** 2026-01-19

**Authors:** Ayush Mittal, Souvik Manna, Vincy Nelson, Nikhilesh Ladha

**Affiliations:** 1 School of Medicine, Employees State Insurance Corporation Medical College and Hospital, Alwar, IND; 2 Community Medicine, Employees State Insurance Corporation Medical College and Hospital, Alwar, IND

**Keywords:** antibiotic resistance genes, crispr-cas system (clustered regularly interspaced short palindromic repeats)-crispr associated system, grna, multidrug-resistant tuberculosis (mdr-tb), rapid diagnostic tests

## Abstract

Background

Multidrug-resistant tuberculosis (MDR-TB) continues to pose a major challenge to TB elimination in India, where drug resistance and delayed diagnosis contribute significantly to ongoing transmission. Clustered Regularly Interspaced Short Palindromic Repeats (CRISPR) based diagnostics have emerged as versatile tools, compared to GeneXpert, capable of detecting resistance-associated mutations with rapid turnaround and high accuracy. This study aimed to design and in silico validate Clustered Regularly Interspaced Short Palindromic Repeats-CRISPR-associated protein (CRISPR-Cas)-based guide RNAs (gRNAs) targeting major drug-resistance mutations in Indian *Mycobacterium tuberculosis* (*M. tuberculosis*) isolates.

Methods

Whole-genome mutation profiles were analyzed using TBProfiler, and gRNAs were designed using CHOPCHOP. Off-target evaluation was performed using Cas-OFFinder and Basic Local Alignment Search Tool (BLAST). High-confidence mutations in *gyrA, rpoB, katG, rpsL, embB, *and* ethA *were selected based on prevalence in Indian isolates and WHO-defined resistance markers.

Results

Numerous drug resistance-associated mutations were identified in the drug-resistant tuberculosis genome isolates. The study identified six key genetic mutations identified in MTB isolates that are associated with phenotypic drug resistance, including *gyrA *(Asp94Gly)*, rpoB *(Ser450Leu)*, *and* katG *(Ser315Thr). For each of the six genes, the chromosome position, locus ID, mutation type, and affected amino acids were identified, and tailored guide RNAs were designed in silico. Top-ranked gRNAs demonstrated optimal GC content, high predicted cleavage efficiency, and zero off-target activity. Each resistance locus yielded multiple candidate gRNAs suitable for CRISPR-based assays.

Conclusions

This computational in silico analysis provides a robust panel of mutation-targeted gRNAs tailored to Indian MDR-TB genomic profiles. These findings lay a strong foundation for developing rapid, affordable CRISPR diagnostics for point-of-care detection of drug resistance. Future laboratory validation and clinical testing are essential for translation into diagnostic practice.

## Introduction

Tuberculosis (TB) remains a major public health challenge in India, with multidrug-resistant tuberculosis (MDR-TB) emerging as a significant barrier to effective treatment. MDR-TB is defined as resistance to at least rifampicin and isoniazid, the two most potent anti-TB drugs. Rapid, accurate, and cost-effective diagnostic tools are critical to improving treatment outcomes and controlling the spread of drug-resistant strains.

Clustered Regularly Interspaced Short Palindromic Repeats-CRISPR-associated protein (CRISPR-Cas) systems have recently emerged as promising tools for molecular diagnostics. The CRISPR-based assays have demonstrated high sensitivity and specificity, achieving near single-copy sensitivity and outperforming traditional methods like culture and GeneXpert in clinical settings [1]. These assays also provide results in a shorter time frame (rapid turnaround), which is essential for timely treatment decisions, and are more cost-effective than the traditional methods [2,3].

Recent studies have explored the potential of CRISPR-based systems for detecting and targeting drug-resistant Mycobacterium tuberculosis. Specifically, the CRISPR-Cas9 system can be guided by RNA molecules (gRNAs) to bind and cleave specific DNA sequences. In silico design of guide RNAs (gRNAs) has been used to target specific genes associated with tuberculosis, such as Rv3378c, which could serve as a potential therapeutic approach [4]. A Cas9/gRNA-assisted quantitative Real-Time PCR assay has also been developed to detect single-nucleotide mutations in the* rpoB* gene, which confer rifampicin resistance [5]. Similarly, a CRISPR-Cas13a system has shown high sensitivity and specificity in detecting fluoroquinolone resistance mutations in MTB [6]. Furthermore, a CRISPR-Cas13a-based diagnostic test for MTB detection in clinical specimens has demonstrated superior sensitivity compared to conventional methods, offering a promising alternative for tuberculosis diagnosis in resource-limited settings [7].

Designing gRNAs that specifically target resistance-conferring mutations in Indian *Mycobacterial tuberculosis* genomes allows for the potential development of highly specific diagnostic assays for the Indian setting.

This project aims to perform an in silico design and validation of gRNAs targeting MDR-associated genes in Indian clinical isolates of *Mycobacterium tuberculosis*. The objectives of this study were to compile and curate a comprehensive database of Indian multidrug-resistant tuberculosis (MDR-TB) genomes, to identify conserved mutations associated with drug resistance, particularly in the *rpoB, katG, inhA, gyrA, rrs, *and *eis* genes, and to evaluate the specificity and potential off-target effects of candidate gRNAs for validation.

## Materials and methods

A descriptive computational study was done from September to November 2025 at ESIC Medical College and Hospital, Alwar, Rajasthan. Ethical approval was waived-off by the Institutional Ethics Committee as the study involved secondary analysis of data available in the public domain.

For data collection, whole genome sequences (WGS) of Indian *M. tuberculosis* isolates were retrieved from the public database of the National Centre for Biotechnology Information (NCBI) [8]. The nucleotide sequences in the FASTQ format, generated using the Illumina HiSeq sequencing system, were downloaded. Isolates were then classified into multidrug-resistant and drug-susceptible groups based on the metadata and resistance profiles available on the NCBI database. One of the most crucial isolates identified during this process was SRR29772703 (SRA ID) with accession number SRX25272822, which was associated with BioProject PRJNA822663. For identification of resistance-associated mutations, web tools like TBProfiler, Mykrobe and MTBseq were used to annotate resistance mutations. The focus was upon high-confidence mutations in key resistance genes as per the WHO catalogue, such as rifampicin resistance (rpoB mutations), isoniazid resistance (katG and inhA mutations), fluoroquinolone resistance (gyrA and gyrB genes) and injectable aminoglycoside resistance (*rrs *and* eis* genes) [9].

Web tools CHOPCHOP and CRISPOR were used to design gRNAs targeting the resistance-conferring mutations. The reference genome version used by the web tools was *Mycobacterium tuberculosis* H37Rv (ASM19595v2). The gRNAs were filtered and selected on the basis of high on-target score, low off-target potential, location (at or near the mutation site), and Protospacer Adjacent Motif (PAM) site availability. Designed gRNAs were tested using the Basic Local Alignment Search Tool (BLAST) for specificity against human and microbial genomes, and using the Off-Target Prediction Tool (Cas-OFFinder) to evaluate potential mismatches in *M. tuberculosis*. Quality check and control of the raw sequence data were performed using FastQC software, and wherever needed, raw reads trimming was done using Trimmomatic software.​​​​

Resistance mutation annotation was done using TBProfiler (version 4.4), which is an open-source bioinformatics tool designed to analyse WGS data of MTB. It predicts drug resistance, identifies strain lineage, and detects large deletions, aiding in tuberculosis surveillance and clinical diagnostics [10]. Next, gRNAs were designed using CHOPCHOP, which is a widely used, web-based tool for designing gRNAs for CRISPR-based genome editing, RNA targeting, and gene regulation. It supports a wide range of CRISPR-associated enzymes and experimental goals. It scans for protospacer adjacent motif (PAM) sequences and generates candidate gRNAs. Off-targets were analysed using mismatch tolerance and gRNAs were scored based on efficiency and specificity [11]. Along with CHOPCHOP, another web tool named CRISPOR was used, which was used to design gRNA with optimum high specificity and on-target efficiency [12].

BLAST was used for comparing an input nucleotide in sequence to a database of known sequences, identifying regions of similarity that indicate functional, structural, or evolutionary relationships [13]. Alongside, the CasOFFinder tool was used for off-target prediction, which is designed to identify potential off-target sites of CRISPR-associated nucleases across entire genomes. It supports an unlimited number of mismatches between the gRNA and potential off-target sites and accommodates various PAM sequences, including degenerate bases, facilitating the analysis of diverse CRISPR systems like SpCas9, SaCas9, and NmCas9 [14].

For quality maintenance, the FastQC tool was used for high-throughput sequencing data, especially data produced by platforms like Illumina. It assesses the quality of raw sequencing reads before downstream analysis like alignment, assembly, or variant calling. Trimmomatic was used for pre-processing Illumina sequencing data, primarily used for adapter clipping, quality filtering, and trimming of reads prior to downstream analyses. It supports both single-end and paired-end read trimming and can be integrated into larger sequencing workflows [15].

## Results

Numerous drug resistance-associated mutations were identified in the polydrug-resistant tuberculosis genome isolates. The study identified the six key genetic mutations identified in *Mycobacterium tuberculosis *isolates that are associated with phenotypic drug resistance. For each of the six genes, the chromosome position, locus ID, mutation type, and affected amino acids were identified from the TBProfiler output. The estimated fraction reflects the proportion of resistant bacterial population carrying that mutation. The corresponding drug class and resistance confidence provide evidence strength linking each mutation to high-level resistance. The study establishes the molecular basis for selecting target genes for CRISPR gRNA design (Table 1).

**Table 1:** Combined result of major genomic mutations associated with drug resistance in Indian Mycobacterium tuberculosis isolates. Source: TBProfiler, *gyrA *gene encodes for A subunit of DNA gyrase, *rpoB* gene encodes for beta subunit (𝛽) of bacterial RNA polymerase, *rpsL* gene encodes for ribosomal protein S12, *katG* gene encodes for catalase-peroxidase enzyme, *embB* gene encodes for arabinosyltransferase responsible for primary resistance to ethambutol, *ethA* encodes for EthA monooxygenase enzyme, which is crucial for activating the antibiotic ethionamide.

Gene	Chromosome position	Locus Position	Mutation	Type	Estimated fraction	Drugs	Confidence	Comment
gyrA	7582	Rv0006	p.Asp94Gly	missense_variant	1	moxifloxacin, levofloxacin	Associated with resistance	High-level resistance
rpoB	761155	Rv0667	p.Ser450Leu	missense_variant	1	rifampicin	Associated with resistance	High-level resistance
rpsL	781687	Rv0682	p.Lys43Arg	missense_variant	0.99	streptomycin	Associated with resistance	-
katG	2155168	Rv1908c	p.Ser315Thr	missense_variant	1	isoniazid	Associated with resistance	High-level resistance
embB	4247429	Rv3795	p.Met306Val	missense_variant	1	ethambutol	Associated with resistance	-
ethA	4326897	Rv3854c	c.-1079_576del	start_lost & conservative_inframe_deletion	1	ethionamide	Associated with resistance	-

Firstly, the study enlisted the top 10 CRISPR guide RNAs out of total 49 candidates found for targeting mutations in the *gyrA *gene, which encodes for A subunit of DNA gyrase and is the cause of primary fluoroquinolone resistance. The parameters recorded were genomic location, GC content, self-complementarity, mismatch tolerance (MM0-MM3), and predicted cutting efficiency. Guides ranked higher show optimal GC% (which should lie between 40% to 60%), minimal secondary structure (which reduces self-complementarity as to minimise self-binding), and zero off-targets, making them suitable for diagnostic applications. The gRNA at rank 1 was designed to be located at chromosome:7110 on the negative strand, and showed the highest efficiency of 73.1% (Table 2).

**Table 2:** gRNAs designed for detection of drug-resistance to fluoroquinolone (RV0006: sequence length 346 BP). GC content (%): percentage of guanine (G) and cytosine (C) in the gRNA sequence; MM0: number of genomic sites with 0 mismatches (perfect match); MM1: number of sites with 1 mismatch; MM2: number of sites with 2 mismatches; MM3: number of sites with 3 mismatches; + strand: gRNA on positive-sense strand; - strand: gRNA on negative-sense strand. Source: CHOPCHOP

Rank	Target sequence	Genomic location	Strand	GC content (%)	Self-complementarity	MM0	MM1	MM2	MM3	Efficiency
1	ACGAACCGAGGGATCCATGGTGG	Chromosome:7110	-	60	0	0	0	0	0	73.1
2	ACAAGGAAGACGGCATTCAGCGG	Chromosome:7044	+	50	1	0	0	0	0	71.82
3	CAGGATGGAGAACAACTCGTCGG	Chromosome:7170	-	50	0	0	0	0	0	69.86
4	GGCCGGGAAGAAGATCAACAAGG	Chromosome:7027	+	55	0	0	0	0	0	69.35
5	GATGGAGAACAACTCGTCGGCGG	Chromosome:7167	-	55	0	0	0	0	0	68
6	TGACGCAACACACGAACCGAGGG	Chromosome:7121	-	55	0	0	0	0	0	63.89
7	TTGACGCAACACACGAACCGAGG	Chromosome:7122	-	55	0	0	0	0	0	63.1
8	GGAGTTGTGGGAGACCACCATGG	Chromosome:7096	+	60	1	0	0	0	0	62.82
9	TGGGCGAGGACGTCGACGCGCGG	Chromosome:7194	+	75	1	0	0	0	0	62.21
10	GGAGACCACCATGGATCCCTCGG	Chromosome:7105	+	60	0	0	0	0	0	60.77

The study also evaluated potential off-target locations for the highest-ranked *gyrA* gRNA. Primer coordinates (both left and right), sequences, melting temperature (Tm, in °C), and off-target counts were enlisted. Zero off-targets across all primer pairs indicated high specificity, which is essential for accurate CRISPR-based detection. The product sizes reflect expected amplicon lengths for downstream validation (Table 3).

**Table 3:** Off-target summary for fluoroquinolone gene gRNA (rank 1). No off-target sequences were identified at any location according to CHOPCHOP; Tm: melting temperature of primer (in degrees celsius). Source: CHOPCHOP.

Pair	Left primer coordinates	Left primer	Left primer Tm	Left primer off-targets	Right primer coordinates	Right primer	Right primer Tm	Right primer off-targets	Pair off-targets	Product size
1	Chromosome:6940-6962	GCTGTACAAACTCAAGTGGCAG	60	0	Chromosome:7173-7195	ATCAGGATGGAGAACAACTCGT	60	0	0	255
2	Chromosome:6941-6963	CTGTACAAACTCAAGTGGCAGC	60	0	Chromosome:7173-7195	ATCAGGATGGAGAACAACTCGT	60	0	0	254
3	Chromosome:7069-7091	CAAGGGTCTAGGTGAAATGGAC	59.9	0	Chromosome:7247-7269	GTTAGACATCCAGGAACCGAAC	59.9	0	0	200
4	Chromosome:7035-7057	AGAAGATCAACAAGGAAGACGG	59.8	0	Chromosome:7173-7195	ATCAGGATGGAGAACAACTCGT	60	0	0	160
5	Chromosome:7068-7090	ACAAGGGTCTAGGTGAAATGGA	59.9	0	Chromosome:7247-7269	GTTAGACATCCAGGAACCGAAC	59.9	0	0	201

**Figure 1:**
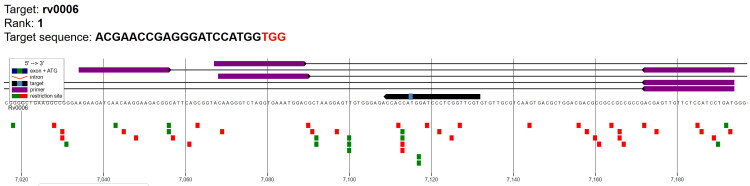
Genome map of gRNA candidate for gyrA with maximum cutting efficiency (rank 1). Target Rv0006 is the gene for *GyrA*, the A subunit of DNA gyrase in *Mycobacterium tuberculosis *genome (Mtb). The purple bars indicate the guide RNA binding region, while the target sequence and PAM motif (shown as a small light blue box on the black arrow) are highlighted along the genomic coordinate. The black arrow illustrates the orientation of the* gyrA* coding sequence. Green squares represent predicted primer binding sites and red squares denote restriction enzyme recognition sites.

This study documented genomic alignment of the highest-ranked CRISPR gRNA targeting *gyrA* (Rv0006). The visual absence of overlapping off-target signals confirms that this (rank 1) gRNA exhibits high genomic specificity suitable for CRISPR-based detection of fluoroquinolone-resistance mutations (Figure 1).

Next, the study enlisted the top 10 CRISPR guide RNAs out of total 51 candidates found for targeting mutations in the* rpoB *gene, which encodes for beta-subunit of RNA polymerase and is the cause of primary rifampicin resistance. These guides specifically target the rifampicin-resistance determining region (RRDR). The gRNA at rank 1 was designed to be located at chromosome: 759714 on the positive strand, and showed the highest cutting efficiency of 70.62% (Table 4).

**Table 4:** gRNAs designed for detection of rifampicin-resistance determining region (RRDR). (Rv0667: sequence length 346 bp). GC content (%): percentage of guanine (G) and cytosine (C) in the gRNA sequence; MM0: number of genomic sites with 0 mismatches (perfect match); MM1: number of sites with 1 mismatch; MM2: number of sites with 2 mismatches; MM3: number of sites with 3 mismatches; + strand: gRNA on positive-sense strand; - strand: gRNA on negative-sense strand. Source: CHOPCHOP.

Rank	Target sequence	Genomic location	Strand	GC content (%)	Self-complementarity	MM0	MM1	MM2	MM3	Efficiency
1	CGAAACCGACAAAATTATCGCGG	Chromosome:759714	+	40	0	0	0	0	0	70.62
2	CTGGGCTCAGACTAAGACCACGG	Chromosome:759635	-	55	0	0	0	0	0	70.14
3	TGGTCGCATGAAGTGCTGGAAGG	Chromosome:759777	+	55	0	0	0	0	0	68.39
4	TCACCCGCCACTTGACACCGTGG	Chromosome:759618	+	65	0	0	0	0	0	64.99
5	GCTCCTCTAAGGGCTCTCGTTGG	Chromosome:759757	+	60	0	0	0	0	0	64.82
6	CGTCCAACAATAGCGCAGGACGG	Chromosome:759570	-	55	0	0	0	0	0	64.65
7	AACGAGAGCCCTTAGAGGAGCGG	Chromosome:759755	-	55	0	0	0	0	0	62.46
8	AGTGCTGGAAGGATGCATCTTGG	Chromosome:759788	+	50	0	0	0	0	0	62.2
9	CCAGCTAGCGCCGATATCCGGGG	Chromosome:759517	+	65	2	0	0	0	0	63.81
10	GAGCCCAGTTTGCGGCTCAGCGG	Chromosome:759650	+	65	2	0	0	0	0	63.19

The study evaluated potential off-target locations for the highest-ranked *rpoB* gRNA. There were no off-targets or mismatches found for the gRNA, and the approximate melting temperature of the primers was around 60 °C (Table 5).

**Table 5:** Off-target summary for rifampicin resistance gRNA (rank 1). No off-target sequences were identified at any location according to CHOPCHOP; Tm: melting temperature of primer (in degrees celsius). Source: CHOPCHOP.

Pair	Left primer coordinates	Left primer		Left primer Tm	Left primer off-targets	Right primer coordinates	Right primer	Right primer Tm	Right primer off-targets	Pair off-targets	Product size
1	Chromosome:759629-759651	TTGACACCGTGGTCTTAGTCTG		60.2	0	Chromosome:759859-759881	GAGTTATTCGAGGAACTTTGCG	60.3	0	0	252
2	Chromosome:759629-759651	TTGACACCGTGGTCTTAGTCTG		60.2	0	Chromosome:759827-759849	ACTAGGACTAGCGGCTGTTTTG	60	0	0	220
3	Chromosome:759630-759652	TGACACCGTGGTCTTAGTCTGA		60.7	0	Chromosome:759827-759849	ACTAGGACTAGCGGCTGTTTTG	60	0	0	219
4	Chromosome:759632-759654	ACACCGTGGTCTTAGTCTGAGC		60.7	0	Chromosome:759827-759849	ACTAGGACTAGCGGCTGTTTTG	60	0	0	217
5	Chromosome:759629-759651	TTGACACCGTGGTCTTAGTCTG		60.2	0	Chromosome:759860-759882	GGAGTTATTCGAGGAACTTTGC	59.3	0	0	253

**Figure 2:**
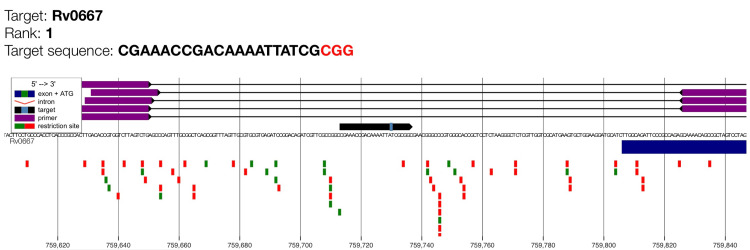
Genome map of gRNA candidate for rpoB with maximum cutting efficiency (rank 1). Target Rv0667 is the *rpoB* gene which encodes for beta subunit (𝛽) of bacterial RNA polymerase. The purple bars indicate the guide RNA binding region, while the target sequence and PAM motif (shown as a small light blue box on the black arrow) are highlighted along the genomic coordinate. The black arrow illustrates the orientation of the *rpoB* coding sequence. Green squares represent predicted primer binding sites and red squares denote restriction enzyme recognition sites. The dark blue rectangular bar represents the initial part of the coding exon region which indicates the position of the guide RNA target relative to the gene structure.

This study also documented the genomic alignment of the highest-ranked CRISPR gRNA targeting* rpoB* (Rv0667). The visual absence of overlapping off-target signals confirms that the rank 1 gRNA exhibits high genomic specificity suitable for CRISPR-based detection of rifampicin-resistance mutations (Figure 2).

Next, the study enlisted the top 10 CRISPR guide RNAs out of total 57 candidates found for targeting mutations in the* rpsL* gene, which encodes for the ribosomal protein S12 and is the cause of primary streptomycin resistance. The gRNA at rank 1 was designed to be located at chromosome:781273 on the positive strand, and showed the highest cutting efficiency at 74.02%, along with zero self-complementarity and 65% GC content (Table 6).

**Table 6:** gRNAs designed for detection of streptomycin-resistance (Rv0682: sequence length 346 bp). GC content (%): percentage of guanine (G) and cytosine (C) in the gRNA sequence; MM0: number of genomic sites with 0 mismatches (perfect match); MM1: number of sites with 1 mismatch; MM2: number of sites with 2 mismatches; MM3: number of sites with 3 mismatches; + strand: gRNA on positive-sense strand; - strand: gRNA on negative-sense strand. Source: CHOPCHOP.

Rank	Target sequence	Genomic location	Strand	GC content (%)	Self-complementarity	MM0	MM1	MM2	MM3	Efficiency
1	GAAAGGCGCACCACACACGGTGG	Chromosome:781273	+	65	0	0	0	0	0	74.02
2	TTGTGGTTGCTCGTGCCTGGCGG	Chromosome:781365	+	60	1	0	0	0	0	69.93
3	TCGTCTCATCCGAGGCATCGCGG	Chromosome:781433	-	60	2	0	0	0	0	70.51
4	AAGCGCCCAAGATAGAAAGCCGG	Chromosome:781534	+	50	0	0	0	0	0	68.22
5	AGCAGGGCGCTACGTTGACGCGG	Chromosome:781298	-	65	1	0	0	0	0	68.16
6	GCTTTGACCTGCCAGACTGGCGG	Chromosome:781336	+	60	2	0	0	0	0	68.26
7	GCAGGTCAAAGCGGGCGTTGCGG	Chromosome:781325	-	65	0	0	0	0	0	64.87
8	AACTCGATTCGTCTCATCCGAGG	Chromosome:781441	-	50	1	0	0	0	0	63.24
9	GGCAAGCTATGCGACACACCCGG	Chromosome:781467	+	60	0	0	0	0	0	61.84
10	GCCAGTCTGGCAGGTCAAAGCGG	Chromosome:781334	-	60	0	0	0	0	0	59.98

Then the study evaluated potential off-target locations for the highest-ranked *rpsL* gRNA, which showed no potential off-targets (Table 7).

**Table 7:** Off-target summary for streptomycin resistance gRNA (rank 1). No off-target sequences were identified at any location according to CHOPCHOP; Tm: melting temperature of primer (in degrees celsius). Source: CHOPCHOP

Pair	Left primer coordinates	Left primer	Left primer Tm	Left primer off-targets	Right primer coordinates	Right primer	Right primer Tm	Right primer off-targets	Pair off-targets	Product size
1	Chromosome:781181-781203	GGTTTGTGTTGCTGGAAATGAC	61.7	0	Chromosome:781447-781469	CCTCAAACTCGATTCGTCTCAT	60.6	0	0	288
2	Chromosome:781182-781204	GTTTGTGTTGCTGGAAATGACC	61.7	0	Chromosome:781447-781469	CCTCAAACTCGATTCGTCTCAT	60.6	0	0	287
3	Chromosome:781182-781204	GTTTGTGTTGCTGGAAATGACC	61.7	0	Chromosome:781446-781468	CTCAAACTCGATTCGTCTCATC	58.9	0	0	286
4	Chromosome:781183-781204	TTTGTGTTGCTGGAAATGACC	60.9	0	Chromosome:781447-781469	CCTCAAACTCGATTCGTCTCAT	60.6	0	0	286
5	Chromosome:781181-781203	GGTTTGTGTTGCTGGAAATGAC	61.7	0	Chromosome:781446-781468	CTCAAACTCGATTCGTCTCATC	58.9	0	0	287

**Figure 3:**
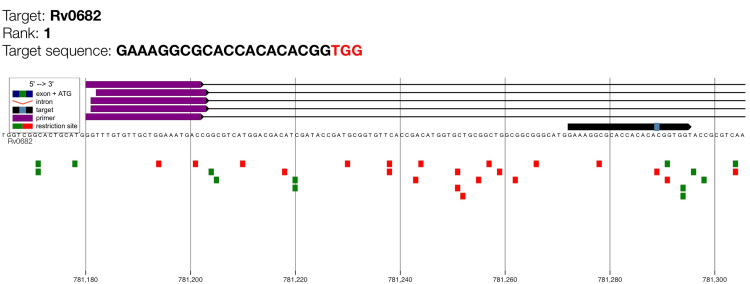
Genome map of gRNA candidate for rpsL with maximum cutting efficiency (rank 1). Target Rv0682 is the* rpsL* gene which encodes for ribosomal protein S12. The purple bars indicate the guide RNA binding region, while the target sequence and PAM motif (shown as a small light blue box on the black arrow) are highlighted along the genomic coordinate. The black arrow illustrates the orientation of the* rpsL* coding sequence. Green squares represent predicted primer binding sites and red squares denote restriction enzyme recognition sites.

This study also depicted a visual representation of the genomic alignment of the highest-ranked CRISPR gRNA targeting *rpsL* (Rv0682). The visual absence of overlapping off-target signals confirms that the rank 1 gRNA exhibits high genomic specificity suitable for CRISPR-based detection of streptomycin-resistance mutations (Figure 3).

Next, the study enlisted the top 10 CRISPR guide RNAs out of total 52 candidates found for targeting mutations, specifically the S315T mutation, in the* katG* gene, which encodes for the catalase peroxidase enzyme and is the cause of primary isoniazid resistance. The gRNA at rank 1 was designed to be located at chromosome:2156269 on the positive strand, and showed the highest cutting efficiency at 66.32%, with GC content within the optimum range (Table 8).

**Table 8:** gRNAs designed for detection of isoniazid-resistance (Rv1908c: sequence length 346 bp). GC content (%): percentage of guanine (G) and cytosine (C) in the gRNA sequence; MM0: number of genomic sites with 0 mismatches (perfect match); MM1: number of sites with 1 mismatch; MM2: number of sites with 2 mismatches; MM3: number of sites with 3 mismatches; + strand: gRNA on positive-sense strand; - strand: gRNA on negative-sense strand. Source: CHOPCHOP.

Rank	Target sequence	Genomic location	Strand	GC content (%)	Self-complementarity	MM0	MM1	MM2	MM3	Efficiency
1	GCCAACAGCACAGTCGACATCGG	Chromosome:2156269	+	55	0	0	0	0	0	66.32
2	CGATAACACCAACTCCTGGAAGG	Chromosome:2156118	-	50	0	0	0	0	0	65.4
3	GGCATGCAGCACGTCGTACACGG	Chromosome:2156407	+	60	0	0	0	0	0	64.26
4	TGTCGACTGTGCTGTTGGCGAGG	Chromosome:2156265	-	60	0	0	0	0	0	63.96
5	CTTTCGCACCAAGCCCGCGGCGG	Chromosome:2156380	+	70	1	0	0	0	0	64.95
6	GATCTTTCGCACCAAGCCCGCGG	Chromosome:2156377	+	60	1	0	0	0	0	64.92
7	AAGACCGGCAGACGATGTGATGG	Chromosome:2156304	+	55	0	0	0	0	0	62.64
8	ACCGGCAGACGATGTGATGGTGG	Chromosome:2156307	+	60	0	0	0	0	0	62.63
9	CATCGTCTGCCGGTCTTGCGGGG	Chromosome:2156298	-	65	0	0	0	0	0	61.77
10	ACCACCATCACATCGTCTGCCGG	Chromosome:2156308	-	55	0	0	0	0	0	61.22

The study evaluated potential off-target locations for the highest-ranked *katG* gRNA, which showed no potential off-targets (Table 9).

**Table 9:** Off-target summary for isoniazid resistance gRNA (rank 1). No off-target sequences were identified at any location according to CHOPCHOP; Tm: melting temperature of primer (in degrees celsius). Source: CHOPCHOP.

Pair	Left primer coordinates	Left primer	Left primer Tm	Left primer off-targets	Right primer coordinates	Right primer	Right primer Tm	Right primer off-targets	Pair off-targets	Product size
1	Chromosome:2156313-2156334	ACAACCACCATCACATCGTCT	60.3	0	Chromosome:2156183-2156205	CAATCAGGACATAGACCCCAGT	60.2	0	0	151
2	Chromosome:2156313-2156334	ACAACCACCATCACATCGTCT	60.3	0	Chromosome:2156118-2156140	CCTTCCAGGAGTTGGTGTTATC	59.9	0	0	216
3	Chromosome:2156313-2156334	ACAACCACCATCACATCGTCT	60.3	0	Chromosome:2156180-2156202	GAACAATCAGGACATAGACCCC	59.7	0	0	154
4	Chromosome:2156313-2156334	ACAACCACCATCACATCGTCT	60.3	0	Chromosome:2156182-2156204	ACAATCAGGACATAGACCCCAG	60.2	0	0	152
5	Chromosome:2156313-2156334	ACAACCACCATCACATCGTCT	60.3	0	Chromosome:2156168-2156190	GTGTCGGATATCGAACAATCAG	59.5	0	0	166

**Figure 4:**
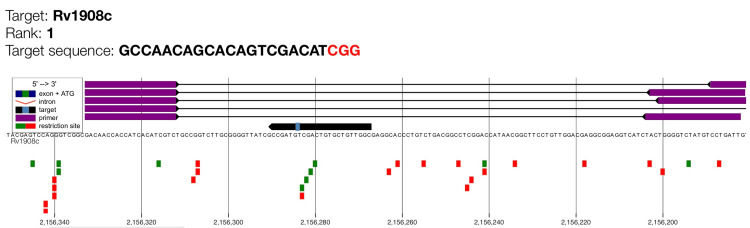
: Genome map of gRNA candidate for katG with maximum cutting efficiency (rank 1). Target Rv1908c is the *katG* gene which encodes for catalase-peroxidase enzyme. The purple bars indicate the guide RNA binding region, while the target sequence and PAM motif (shown as a small light blue box on the black arrow) are highlighted along the genomic coordinate. The black arrow illustrates the orientation of the *katG* coding sequence. Green squares represent predicted primer binding sites and red squares denote restriction enzyme recognition sites.

This study documented the genomic alignment of the highest-ranked CRISPR gRNA targeting *katG* (Rv1908c), the principal gene associated with isoniazid resistance. The visual absence of overlapping off-target signals confirms that this (rank 1) gRNA exhibits high genomic specificity suitable for CRISPR-based detection of isoniazid-resistance mutations (Figure 4).

Next, the study enlisted the top 10 CRISPR guide RNAs out of total 66 candidates found for targeting mutations, specifically the codon 306 mutation, in the *embB* gene, which encodes for the arabinosyltransferase enzyme and is the cause of primary ethambutol resistance. The gRNA at rank 1 was designed to be located at chromosome:4246447 on the negative strand, and showed the highest cutting efficiency at 72.3% (Table 10).

**Table 10:** gRNAs designed for detection of ethambutol-resistance (Rv3795: sequence length 346 bp). GC content (%): percentage of guanine (G) and cytosine (C) in the gRNA sequence; MM0: number of genomic sites with 0 mismatches (perfect match); MM1: number of sites with 1 mismatch; MM2: number of sites with 2 mismatches; MM3: number of sites with 3 mismatches; + strand: gRNA on positive-sense strand; - strand: gRNA on negative-sense strand. Source: CHOPCHOP.

Rank	Target sequence	Genomic location	Strand	GC content (%)	Self-complementarity	MM0	MM1	MM2	MM3	Efficiency
1	GATCACGCCCTCCTCGACAACGG	Chromosome:4246447	-	60	0	0	0	0	0	72.3
2	GCCGGACCACAAGCAGACGGCGG	Chromosome:4246256	+	70	2	0	0	0	0	67.79
3	GGTGAACAGGAACGGACCGCCGG	Chromosome:4246309	-	65	0	0	0	0	0	64.37
4	CAGGTACGTGGCGATCGTCGAGG	Chromosome:4246348	-	65	0	0	0	0	0	64.1
5	CGCGATACCAGTCCCCACGCAGG	Chromosome:4246367	-	70	2	0	0	0	0	64.03
6	GATCGCCACGTACCTGCGTGGGG	Chromosome:4246355	+	65	1	0	0	0	0	62.8
7	CGGTCCGTTCCTGTTCACCCAGG	Chromosome:4246313	+	65	0	0	0	0	0	59.37
8	CGATCGCCACGTACCTGCGTGGG	Chromosome:4246354	+	65	0	0	0	0	0	58.02
9	GTATCGCGACTGGGGATCGGTGG	Chromosome:4246382	+	65	1	0	0	0	0	59.01
10	CCTGCCGGACCACAAGCAGACGG	Chromosome:4246253	+	65	3	0	0	0	0	60.29

Then the study evaluated potential off-target locations for the highest-ranked *embB* gRNA, which showed no potential off-targets (Table 11).

**Table 11:** Off-target summary for ethambutol resistance gRNA (rank 1). No off-target sequences were identified at any location according to CHOPCHOP; Tm: melting temperature of primer (in degrees celsius). Source: CHOPCHOP.

Pair	Left primer coordinates	Left primer	Left primer Tm	Left primer off-targets	Right primer coordinates	Right primer	Right primer Tm	Right primer off-targets	Pair off-targets	Product size
1	Chromosome:4246395-4246414	GGATCGGTGGAGCAGTACC	60.5	0	Chromosome:4246615-4246636	GACAACACAAAGCCAATCAGC	60.7	0	0	241
2	Chromosome:4246396-4246415	GATCGGTGGAGCAGTACCA	59.6	0	Chromosome:4246615-4246636	GACAACACAAAGCCAATCAGC	60.7	0	0	240
3	Chromosome:4246400-4246418	GGTGGAGCAGTACCACCG	60.1	0	Chromosome:4246615-4246636	GACAACACAAAGCCAATCAGC	60.7	0	0	236
4	Chromosome:4246396-4246415	GATCGGTGGAGCAGTACCA	59.6	0	Chromosome:4246619-4246639	ACCGACAACACAAAGCCAAT	60.4	0	0	243
5	Chromosome:4246364-4246382	GTACCTGCGTGGGGACTG	60.1	0	Chromosome:4246615-4246636	GACAACACAAAGCCAATCAGC	60.7	0	0	272

**Figure 5:**
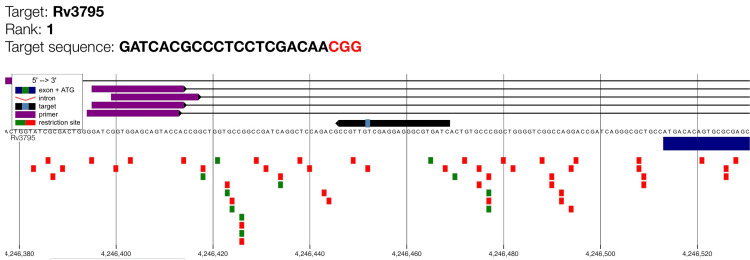
: Genome map of gRNA candidate for embB with maximum cutting efficiency (rank 1). Target Rv3795 is the *embB* gene which encodes for arabinosyltransferase enzyme. The purple bars indicate the guide RNA binding region, while the target sequence and PAM motif (shown as a small light blue box on the black arrow) are highlighted along the genomic coordinate. The black arrow illustrates the orientation of the *embB* coding sequence. Green squares represent predicted primer binding sites and red squares denote restriction enzyme recognition sites. The dark blue rectangular bar represents the initial part of the coding exon region which indicates the position of the guide RNA target relative to the gene structure.

This study also documented the genomic alignment of the highest-ranked CRISPR gRNA targeting *embB* (Rv3795), the principal gene associated with ethambutol resistance. The visual absence of overlapping off-target signals confirms that the rank 1 gRNA exhibits high genomic specificity suitable for CRISPR-based detection of ethambutol-resistance mutations (Figure 5).

Next, the study enlisted the top 10 CRISPR guide RNAs out of total 55 candidates found for targeting mutations in the promoter and coding regions of the *ethA *gene, which encodes for the EthA mono-oxygenase enzyme, and is the cause of primary ethionamide resistance. The gRNA at rank 1 was designed to be located at chromosome:4327669 on the negative strand, and showed the highest efficiency at 74.34%, with 1 location of self-complementarity (Table 12).

**Table 12:** gRNAs designed for detection of ethionamide-resistance (Rv3854c: sequence length 346 bp). GC content (%): percentage of guanine (G) and cytosine (C) in the gRNA sequence; MM0: number of genomic sites with 0 mismatches (perfect match); MM1: number of sites with 1 mismatch; MM2: number of sites with 2 mismatches; MM3: number of sites with 3 mismatches; + strand: gRNA on positive-sense strand; - strand: gRNA on negative-sense strand. Source: CHOPCHOP.

Rank	Target sequence	Genomic location	Strand	GC content (%)	Self-complementarity	MM0	MM1	MM2	MM3	Efficiency
1	TCGACCGAGATATCGGCCAGCGG	Chromosome:4327669	-	60	1	0	0	0	0	74.34
2	GGGAAATAGAAGTAGAACGTCGG	Chromosome:4327723	-	40	0	0	0	0	0	71.96
3	GTCCTCGAGAAGGTTCTCGGCGG	Chromosome:4327643	-	60	2	0	0	0	0	73.21
4	CACCGCCGAGAACCTTCTCGAGG	Chromosome:4327641	+	65	2	0	0	0	0	68.63
5	TCGGTCGACGATCTGGCCAAGGG	Chromosome:4327684	+	60	0	0	0	0	0	65.51
6	ACGCTATCAACGTAATGTCGAGG	Chromosome:4327480	+	45	0	0	0	0	0	65.29
7	TCGACATTACGTTGATAGCGTGG	Chromosome:4327478	-	45	0	0	0	0	0	64.82
8	AGTCAGGCTTCGCTGCCTAGGGG	Chromosome:4327567	+	60	2	0	0	0	0	66.52
9	ATTGACCACCCGGTCCAGCAGGG	Chromosome:4327769	-	60	2	0	0	0	0	65.8
10	CGACACGTAGTAAGCTGCCAGGG	Chromosome:4327527	+	55	0	0	0	0	0	63.62

The current study evaluated potential off-target locations for the highest-ranked* ethA* gRNA, which showed no potential off-targets (Table 13).

**Table 13:** Off-target summary for ethionamide resistance gRNA (rank 1). No off-target sequences were identified at any location according to CHOPCHOP; Tm: melting temperature of primer (in degrees celsius). Source: CHOPCHOP.

Pair	Left primer coordinates	Left primer	Left primer Tm	Left primer off-targets	Right primer coordinates	Right primer	Right primer Tm	Right primer off-targets	Pair off-targets	Product size
1	Chromosome:4327735-4327757	CTTCCTTGGATGGGAAATAGAA	59.4	0	Chromosome:4327477-4327499	TCCACGCTATCAACGTAATGTC	60	0	0	280
2	Chromosome:4327734-4327756	TTCCTTGGATGGGAAATAGAAG	59.4	0	Chromosome:4327477-4327499	TCCACGCTATCAACGTAATGTC	60	0	0	279
3	Chromosome:4327735-4327757	CTTCCTTGGATGGGAAATAGAA	59.4	0	Chromosome:4327474-4327496	GGATCCACGCTATCAACGTAAT	60.2	0	0	283
4	Chromosome:4327734-4327756	TTCCTTGGATGGGAAATAGAAG	59.4	0	Chromosome:4327474-4327496	GGATCCACGCTATCAACGTAAT	60.2	0	0	282
5	Chromosome:4327734-4327756	TTCCTTGGATGGGAAATAGAAG	59.4	0	Chromosome:4327475-4327497	GATCCACGCTATCAACGTAATG	59.5	0	0	281

**Figure 6:**
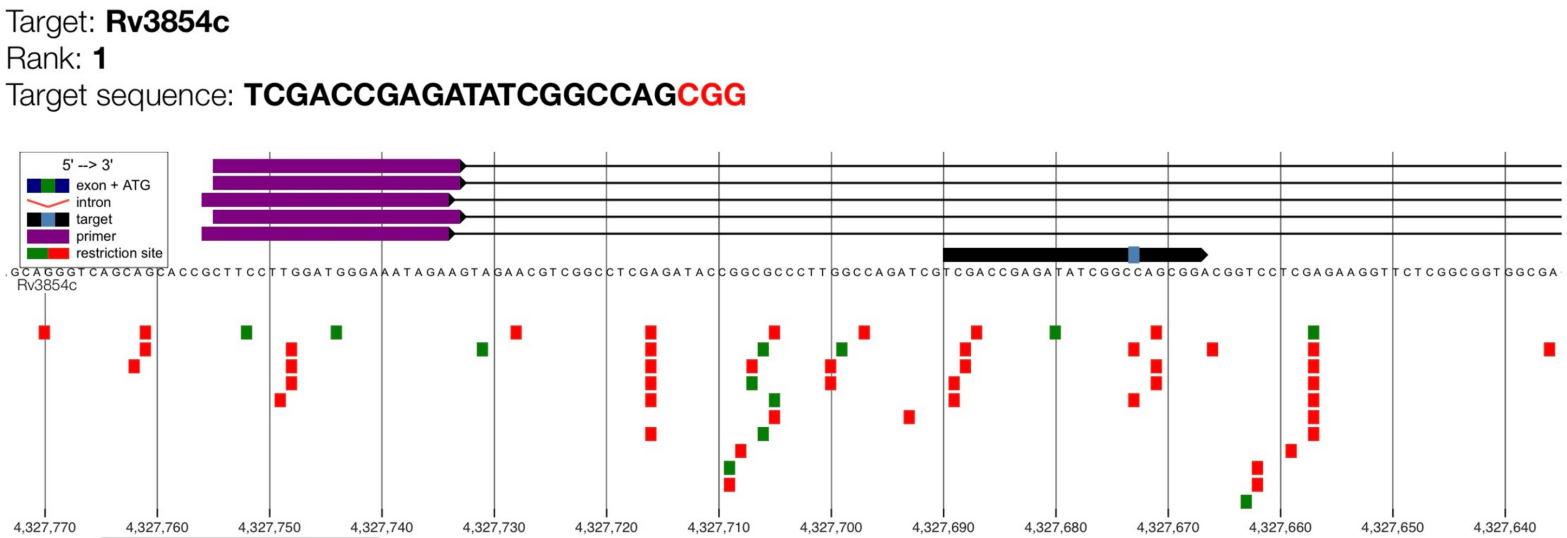
Genome map of gRNA candidate for ethA with maximum cutting efficiency (rank 1). Target Rv3854c is the *ethA* gene which encodes for EthA mono-oxygenase enzyme. The purple bars indicate the guide RNA binding region, while the target sequence and PAM motif (shown as a small light blue box on the black arrow) are highlighted along the genomic coordinate. The black arrow illustrates the orientation of the *ethA* coding sequence. Green squares represent predicted primer binding sites and red squares denote restriction enzyme recognition sites.

This study documented the genomic alignment of the highest-ranked CRISPR gRNA targeting *ethA* (Rv3854c). The visual absence of overlapping off-target signals confirms that the rank 1 gRNA exhibits high genomic specificity suitable for CRISPR-based detection of ethionamide-resistance mutations (Figure 6).

## Discussion

The present in silico study demonstrates the feasibility and robustness of using CRISPR-based gRNA design for detecting multidrug-resistant *Mycobacterium tuberculosis* in the Indian setting. Using genomic mutation profiles from TBProfiler and gRNA design via CHOPCHOP, we successfully identified six high-confidence resistance mutations and generated highly specific corresponding gRNAs with zero off‑target activity. The *gyrA* (Asp94Gly) mutation, which is strongly associated with high-level fluoroquinolone resistance, has been widely reported in Indian and international genomic surveillance studies [16]. Likewise, the *rpoB* (Ser450Leu) mutation, the most common mutation conferring rifampicin resistance worldwide, was present with 100% estimated fraction in the analysed isolate, consistent with prior epidemiological datasets [17]. The *katG* (Ser315Thr) mutation, a hallmark of isoniazid resistance in both high- and low-burden settings, has been extensively documented [17]. The gRNAs designed in this study displayed excellent predicted diagnostic performance, characterized by zero off-target hits, optimal GC content, and high predicted SpCas9/Cas12a cutting efficiency. These characteristics are essential for CRISPR diagnostics such as SHERLOCK (Specific High-sensitivity Enzymatic Reporter unLOCKing) and DETECTR (DNA Endonuclease Targeted CRISPR Trans Reporter), where even minor off-target cleavage can result in false‑positive or ambiguous signals. Similar CRISPR-based approaches, such as Cas13a assays for fluoroquinolone resistance, have demonstrated exceptional sensitivity and SNP (Single Nucleotide Polymorphism) level specificity [18]. CRISPR diagnostics have already shown superior sensitivity compared to GeneXpert in clinical studies [1]. The region‑specific gRNA design used here, therefore, has the potential to significantly improve diagnostic accuracy for Indian MTB lineages, which are known to harbour unique mutation spectra [19]. Rapid diagnosis may even be more beneficial in the risk group, which is non-adherent to treatment or at a higher risk of developing tuberculosis infection [20,21]. Previous studies have also demonstrated that there is low awareness about MDR-TB and it has a higher burden among vulnerable populations, like tribals, diabetics, immunosuppressed, etc [22-24].

There are six key mutations conferring resistance to the mycobacteria. For example, essentiality screens based on saturation mutagenesis have identified the Rv0006 mutation among the loci required for in vitro viability, suggesting strong functional constraints on the region, leading to rifampicin resistance [25,26].

During prospective wet-lab validation, the target sequence may be embedded across exon-annotated regions without intronic interruption, consistent with the fact that *M. tuberculosis* genes are typically intron-less. Primer design tools such as Primer3 and related engines use similar positional penalty plotting to display local sequence constraints, including secondary structure, GC micro-domains, and homopolymer runs, all of which influence binding efficiency [27]. The restriction enzyme recognition site, at the annotated target site, is a common feature included when designing amplicons for downstream cloning or genotyping assays. Restriction-site placement at the location suggests that the selected fragment provides diagnostic utility while maintaining compatibility with the surrounding reading frame, an important principle when designing constructs intended for recombinant analysis or allelic differentiation [28]. Certain regions, especially those with elevated GC content, typical of the *M. tuberculosis* genome, may pose limitations for oligonucleotide binding. MTB genes average ~65% GC content, which is known to impose challenges for primer annealing and secondary-structure avoidance [29]. This pattern aligns with published analyses on GC-rich pathogen genomes, where primer and probe performance strongly depends on micro-scale GC patterns, stable hairpins, and repetitive motifs [30]. Although the study demonstrates strong computational evidence supporting these gRNAs, experimental validation is essential to confirm their real‑world performance, considering factors such as secondary DNA structure, nuclease kinetics, and biological matrix effects. Nonetheless, the computational pipeline developed here provides a strong foundation for the future development of CRISPR‑based MDR‑TB diagnostics.

Implications

The results of this study carry significant implications for MDR-TB diagnostics in India. The gRNAs designed and validated in silico can be directly incorporated into CRISPR diagnostic platforms, including SHERLOCK and DETECTR assays. Their mutation‑specific detection capability offers rapid and accurate differentiation between drug-sensitive and drug-resistant strains, which is critical for early treatment initiation. By focusing on Indian mutation patterns, this panel has the potential to improve diagnostic sensitivity in regional populations, supporting the National TB Elimination Programme (NTEP), which aims to eliminate TB from India by 2030. The low cost, portability, and rapid turnaround of CRISPR assays make them ideal for deployment in district-level laboratories. Additionally, multiplexing these gRNAs could enable simultaneous detection of resistance to multiple drugs, strengthening surveillance for MDR and pre-XDR-TB. Beyond diagnostics, this gRNA panel may also inform future CRISPR-based therapeutic research, although translation into treatment will require extensive biosafety and efficacy studies.

Limitations

This study is subject to several limitations. As an in silico analysis, the results rely on computational predictions and publicly available genomic datasets, which may not fully represent the genetic diversity of *Mycobacterium tuberculosis* across India. Rare or lineage‑specific resistance mutations may, therefore, be under-represented. Although all gRNAs demonstrated zero predicted off-target interactions, in vitro biochemical performance can differ due to DNA accessibility, secondary structure, nuclease kinetics, and sample quality. Thus, laboratory validation remains essential. CRISPR diagnostics also face practical challenges such as contamination risk, reagent stability, and workflow standardization which were beyond the scope of this analysis. Additionally, the study focused primarily on high-confidence mutations; compensatory or rare variants contributing to drug resistance were not included. Therefore, future expansion of the gRNA panel is recommended for more comprehensive coverage.

## Conclusions

This study successfully designed and evaluated, in silico, a panel of CRISPR-based gRNAs targeting major MDR‑TB resistance mutations found in Indian *Mycobacterium tuberculosis *isolates. The detection of hallmark mutations such as *gyrA* (Asp94Gly), *rpoB *(Ser450Leu), and *katG* (Ser315Thr) reinforces the relevance of the mutation set used. The designed gRNAs exhibit strong predicted specificity, zero off‑target activity, and high cutting efficiency, making them strong candidates for CRISPR diagnostic applications in India. These findings provide foundational work toward developing rapid, affordable, mutation‑specific point‑of‑care diagnostics that can support India’s TB elimination goals. Future laboratory studies and clinical validation will be essential for translating these in silico predictions into practical diagnostic tools.
